# The Abnormal Phenotypes of Cartilage and Bone in Calcium-Sensing Receptor Deficient Mice Are Dependent on the Actions of Calcium, Phosphorus, and PTH

**DOI:** 10.1371/journal.pgen.1002294

**Published:** 2011-09-22

**Authors:** Jingning Liu, Fangqiao Lv, Wen Sun, Chunxiang Tao, Guoxian Ding, Andrew Karaplis, Edward Brown, David Goltzman, Dengshun Miao

**Affiliations:** 1 State Key Laboratory of Reproductive Medicine, The Research Center for Bone and Stem Cells, Department of Anatomy, Histology, and Embryology, Nanjing Medical University, Nanjing, China; 2 Institute of Dental Research, Stomatological College, Nanjing Medical University, Nanjing, China; 3 Department of Gerontology, First Affiliated Hospital of Nanjing Medical University, Nanjing, China; 4 Department of Medicine, McGill University, Montreal, Canada; 5 Division of Endocrinology, Diabetes, and Hypertension, Department of Medicine, Brigham and Women's Hospital, Harvard Medical School, Boston, Massachusetts, United States of America; University of Washington, United States of America

## Abstract

Patients with neonatal severe hyperparathyroidism (NSHPT) are homozygous for the calcium-sensing receptor (CaR) mutation and have very high circulating PTH, abundant parathyroid hyperplasia, and severe life-threatening hypercalcemia. Mice with homozygous deletion of CaR mimic the syndrome of NSHPT. To determine effects of CaR deficiency on skeletal development and interactions between CaR and 1,25(OH)_2_D_3_ or PTH on calcium and skeletal homeostasis, we compared the skeletal phenotypes of homozygous CaR–deficient (CaR^−/−^) mice to those of double homozygous CaR– and 1α(OH)ase–deficient [CaR^−/−^1α(OH)ase^−/−^] mice or those of double homozygous CaR– and PTH–deficient [CaR^−/−^PTH^−/−^] mice at 2 weeks of age. Compared to wild-type littermates, CaR^−/−^ mice had hypercalcemia, hypophosphatemia, hyperparathyroidism, and severe skeletal growth retardation. Chondrocyte proliferation and PTHrP expression in growth plates were reduced significantly, whereas trabecular volume, osteoblast number, osteocalcin-positive areas, expression of the ALP, type I collagen, osteocalcin genes, and serum ALP levels were increased significantly. Deletion of 1α(OH)ase in CaR^−/−^ mice resulted in a longer lifespan, normocalcemia, lower serum phosphorus, greater elevation in PTH, slight improvement in skeletal growth with increased chondrocyte proliferation and PTHrP expression, and further increases in indices of osteoblastic bone formation. Deletion of PTH in CaR^−/−^ mice resulted in rescue of early lethality, normocalcemia, increased serum phosphorus, undetectable serum PTH, normalization in skeletal growth with normal chondrocyte proliferation and enhanced PTHrP expression, and dramatic decreases in indices of osteoblastic bone formation. Our results indicate that reductions in hypercalcemia play a critical role in preventing the early lethality of CaR^−/−^ mice and that defects in endochondral bone formation in CaR^−/−^ mice result from effects of the marked elevation in serum calcium concentration and the decreases in serum phosphorus concentration and skeletal PTHrP levels, whereas the increased osteoblastic bone formation results from direct effects of PTH.

## Author Summary

Mice with homozygous deletion of the calcium-sensing receptor (CaR) mimic the syndrome of neonatal severe hyperparathyroidism (NSHPT) in humans with very high circulating parathyroid hormone (PTH) and severe life-threatening hypercalcemia. To determine effects of CaR deficiency on skeletal development and interactions between CaR and 1,25(OH)_2_D_3_ or PTH on calcium and skeletal homeostasis, we compared the skeletal phenotypes of homozygous CaR–deficient mice to those of double homozygous CaR– and 1,25(OH)_2_D_3_–deficient mice or those of double homozygous CaR– and PTH–deficient mice. CaR–deficient mice had hypercalcemia, hypophosphatemia, hyperparathyroidism, severe skeletal growth retardation, and abnormalities; and most died within 2 weeks of age. Deletion of 1,25(OH)_2_D_3_ in CaR–deficient mice resulted in a longer lifespan, normocalcemia, lower serum phosphorus, greater elevation in PTH, and slight improvement in skeletal growth. Deletion of PTH in CaR–deficient mice resulted in rescue of early lethality, normocalcemia, increased serum phosphorus, and normalization in skeletal growth. Our results indicate that reductions in hypercalcemia reduce the early lethality of CaR–deficient mice and that deletion of PTH in patients with NSHPT may normalize skeletal growth and development.

## Introduction

The extracellular calcium-sensing receptor (CaR) is a G protein-coupled receptor that plays an essential role in the regulation of extracellular calcium homeostasis. This receptor is expressed in nearly all tissues [1]. Cloning of the CaR was immediately followed by the association of human genetic diseases with inactivating or activating CaR mutations: familial hypocalciuric hypercalcemia (FHH) and neonatal severe hyperparathyroidism (NSHPT) are caused by CaR-inactivating mutations, whereas autosomal dominant hypoparathyroidism is secondary to CaR-activating mutations [1]. Patients with FHH are heterozygous for the CaR mutation and have a normal or mildly increased circulating parathyroid hormone (PTH) level, normal parathyroid histology or mild parathyroid hyperplasia, and mild to moderate hypercalcemia [2], [3]. Patients with NSHPT are homozygous for the CaR mutation and have very high circulating PTH, abundant parathyroid hyperplasia and severe life-threatening hypercalcemia [4]. Targeted inactivation of the CaR gene in mice has resulted in the development of models of the human syndromes [5]. Thus mice with heterozygous deletion of CaR mimic the syndrome of FHH, whereas homozygotes mimic the syndrome of NSHPT and generally die within a few days to weeks after birth.

A wide variety of functions have been attributed to CaR. Previous studies confirmed that most CaR^−/−^ mice died within 2 weeks after birth [6]. Compared to WT littermates, body size and body weight were reduced markedly, serum calcium levels were markedly elevated, serum phosphorus levels were decreased, serum PTH levels were raised significantly in 2-week-old CaR^−/−^mice, and the parathyroid glands were also enlarged. CaR^−/−^ mice die shortly after birth because of the effects of severe hyperparathyroidism and hypercalcemia. However, the lethal CaR–deficient phenotype has made it difficult to dissect direct effects of CaR deficiency from secondary effects of hyperparathyroidism and hypercalcemia.

Recently, targeted deletion of the CaR from chondrocytes has been reported to be lethal in utero in mice before embryonic day 13 but to produce viable mice with delayed growth plate development if conditional targeted deletion in these cells is induced between E16 and E18 [7]. Targeted deletion of the CaR from early committed osteoblasts resulted in smaller, undermineralized skeletons with significant reductions in bone volume and bone mineral density in the femur and vertebrae [7]. This study demonstrated a critical role for the CaR in skeletal development. However, previous studies failed to find such role for CaR in mice with targeted disruption of exon 5 of the CaR gene encoding a portion of the extracellular domain of this receptor [5], [6]. It has not been established that CaR has a direct role in the skeleton based on this animal model. As noted earlier, these observations may be confounded by the severe hyperparathyroidism and the accompanying hypercalcemia and hypophosphatemia in this animal model. To better understand direct effects of CaR on bone and cartilage function, correction of hyperparathyroidism is required in this CaR–deficient mouse model. We have previously reported a mouse model deficient in PTH by targeting the *Pth* gene in embryonic stem cells [8], [9]. Although adult *Pth*-null mice develop hypocalcemia, hyperphosphatemia and low circulating 1,25(OH)_2_D_3_ levels consistent with primary hypoparathyroidism [9], this phenotype is not lethal. Therefore, a double-knockout model was established by crossing the PTH-deficient with the CaR–deficient mice [10] to correct the severe hyperparathyroidism, hypercalcemia and hypophosphatemia observed in the homozygous CaR–deficient mice. The results of this study indicated that elimination of hyperparathyroidism rescued the increased neonatal mortality as well as the rickets-like skeletal abnormality in these mice suggesting that the early lethality could be corrected by eliminating the hypercalcemia and hyperparathyroidism [10], [11]. However, any essential, nonredundant role for CaR in regulating chondrogenesis or osteogenesis could not be identified based on analysis of the skeleton of this adult double knockout model. It is unclear why there are discrepancies between the findings from CaR conditional knockout mice [7] and those from CaR conventional knockout mice [6], [10] regarding the physiological significance of the CaR in the skeleton, although residual biological activity of an alternatively spliced CaR lacking exon 5 in the conventional, global knockout model may be one explanation.

The vitamin D-PTH axis plays a central role in calcium and phosphate homeostasis and is essential for skeletal development and mineralization. PTH and 1,25-dihydroxyvitamin D_3_ [1,25(OH)_2_D_3_] directly affect calcium homeostasis and each exerts important regulatory effects on the other. PTH stimulates the production of 1,25(OH)_2_D_3_ by activating the renal 25-hydroxyvitamin D-1α-hydroxylase [1α(OH)ase] [12], [13]; 1,25(OH)_2_D_3_, in turn, suppresses the production of PTH [14], [15] and controls parathyroid cell growth [16]. 1,25(OH)_2_D_3_ suppression of PTH synthesis occurs through negative regulation of PTH gene transcription by a 1,25(OH)_2_D_3_-vitamin D receptor (VDR)/retinoid X receptor (RXR) complex [17] in the parathyroid cells [18]. We [19] and others [20] have previously reported a mouse model deficient in 1,25(OH)_2_D by targeted ablation of the 1α(OH)ase gene [1α(OH)ase^−/−^]. After being weaned, mice fed a diet of regular mouse chow developed secondary hyperparathyroidism, retarded growth and skeletal abnormalities characteristic of rickets. These abnormalities mimic those described in vitamin D-dependent rickets type I [21] and this mouse phenotype is not lethal. Furthermore, by comparing mice with targeted disruption of the PTH or 1α(OH)ase genes with PTH-1α(OH)ase double null mice, we found that PTH^−/−^1α(OH)ase^−/−^ mice died of tetany with severe hypocalcemia by 3 weeks of age with severe defects in skeletal development [22].

To determine effects of CaR deficiency on skeletal development as well as to investigate interactions between CaR, 1,25(OH)_2_D_3_ and PTH on calcium and skeletal homeostasis, we compared the skeletal phenotypes of CaR^−/−^ mice to those of the double homozygous, CaR- and 1α(OH)ase-deficient [CaR^−/−^1α(OH)ase^−/−^] mice or those of the double homozygous CaR- and PTH-deficient [CaR^−/−^PTH^−/−^] mice at 2 weeks of age.

## Results

### Effects of deletion of 1α(OH)ase or PTH on lifespan and body weight in CaR–deficient mice

**Figure 1:**
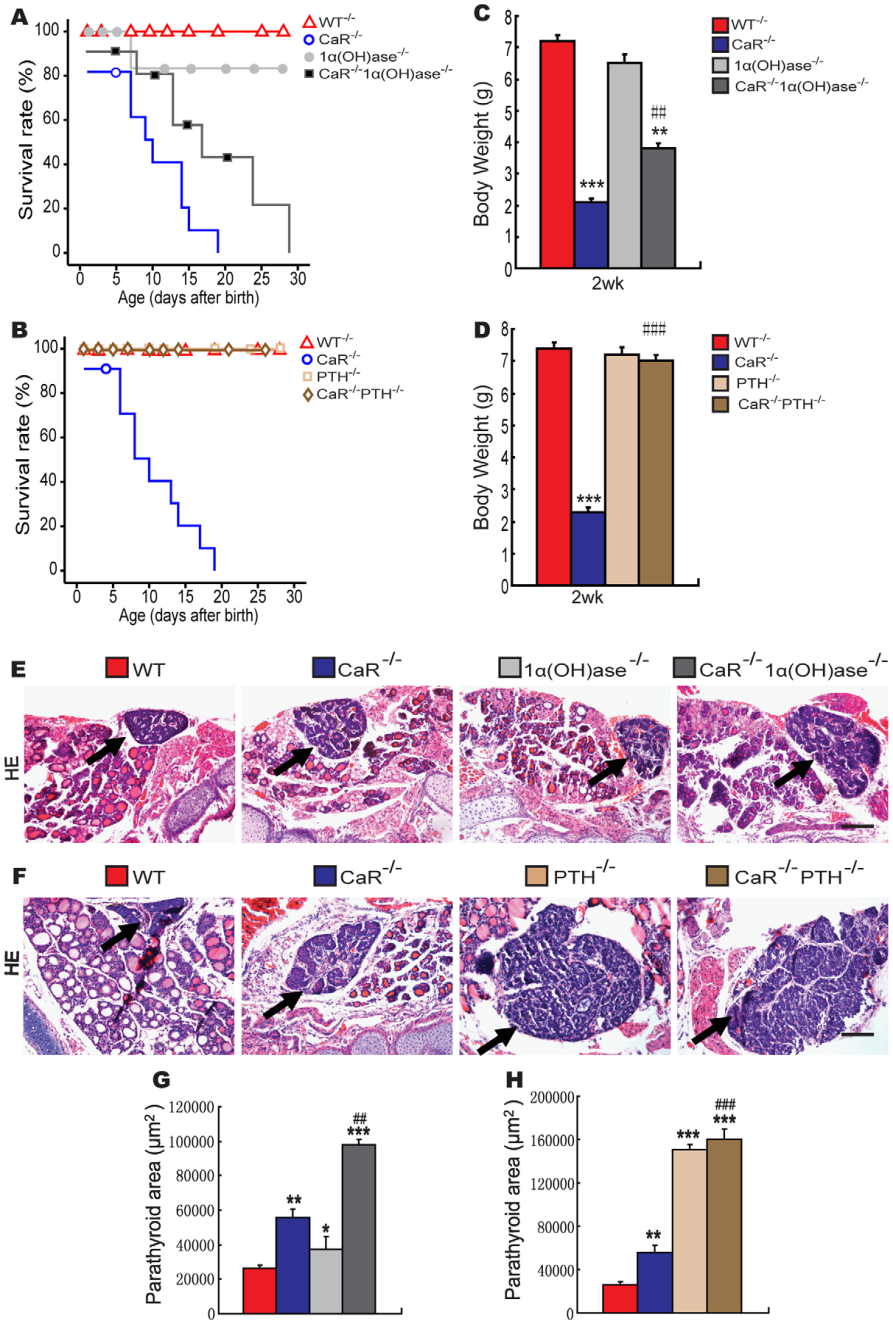
Effects of deletion of 1α(OH)ase or PTH on lifespan, body weight, and parathyroid gland size in CaR–deficient mice. (A) Lifespan of CaR^−/−^ mice with varying 1α(OH)ase status. (B) Lifespan of CaR^−/−^ mice with varying PTH status. (C) Body weight of CaR^−/−^ mice with varying 1α(OH)ase status at 2 weeks of age. (D) Body weight of CaR^−/−^ mice with varying PTH status at 2 weeks of age. (E, F) Representative micrographs of sections of parathyroid glands (arrows) and adjacent thyroid tissues stained with hematoxylin and eosin (HE). Scale bars in K and L represent 100 µm. (G, H) Parathyroid areas. Each value is the mean±SEM of determinations in 6 mice of the same genotype. *, P<0.05; **, P<0.01; ***, P<0.001 compared with wild-type mice. ##, P<0.01; ###, P<0.001 compared with CaR^−/−^ mice.

In CaR^−/−^ mice, the survival rate was 20% and the body weight was decreased significantly at 2 weeks of age; Ablation of 1α(OH)ase in CaR^−/−^ mice resulted in a slightly longer lifespan and a significant increase of body weight; Ablation of PTH in CaR^−/−^ mice resulted in a normalized lifespan and body weight (Figure 1A–1D).

### Effects of deletion of 1α(OH)ase or PTH on serum biochemistries and on parathyroid gland size in CaR^−/−^ mice

At 2 weeks of age, CaR^−/−^ mice displayed hypercalcemia, hypophosphatemia, and increased serum ALP, PTH and 1,25(OH)_2_D_3_; 1α(OH)ase^−/−^ mice displayed mild hypocalcemia, hypophosphatemia, hyperparathyroidism, and undetectable serum 1,25(OH)_2_D_3_; PTH^−/−^ mice had moderate hypocalcemia and hyperphosphatemia, lower serum 1,25(OH)_2_D_3_ and undetectable serum PTH; Ablation of 1α(OH)ase in CaR^−/−^ mice resulted in normocalcemia, more severe hypophosphatemia, and more marked elevations in serum ALP and PTH; In contrast, ablation of PTH in CaR^−/−^ mice resulted in normocalcemia, less severe hyperphosphatemia, normal serum ALP levels,lower serum 1,25(OH)_2_D_3_ and undetectable serum PTH (Table 1).

**Table 1:** Effects of deletion of 1α(OH)ase or PTH on serum biochemistries in CaR^−/−^ mice.

	WT	CaR^−/−^	1α(OH)ase^−/−^	CaR^−/−^1α(OH)ase^−/−^
Serum calcium (mmol/L)	2.7±0.07	3.9±0.22***	2.4±0.05*	2.6±0.08^###^
Serum phosphorus (mmol/L)	3.3±0.14	1.4±0.20***	2.7±0.18*	1.0±0.05*** ^##^
Serum ALP (IU/L)	304±55.5	1880±203***	461±15.3*	2240±44.1*** ^#^
Serum PTH (pg/ml)	18.4±5.02	311±11.4***	28.1±3.34*	492±30.2*** ^##^
Serum 1,25(OH)_2_D_3_ (pg/ml)	158±12.3	314±32.5***	0	0

	WT	CaR^−/−^	PTH^−/−^	CaR^−/−^PTH^−/−^
Serum calcium (mmol/L)	2.5±0.06	4.2±0.20***	1.6±0.12**	2.2±0.11^###^
Serum phosphorus (mmol/L)	3.6±0.14	1.5±0.23***	4.5±0.17**	4.1±0.19* ^###^
Serum ALP (IU/L)	294±48.8	1820±199***	363±15.3	361±22.9^###^
Serum PTH (pg/ml)	20.1±4.23	323±15.8***	0	0
Serum 1,25(OH)_2_D_3_ (pg/ml)	154±9.81	322±28.9***	128±18.2	98.4±21.3** ^###^

*, P<0.05;

**, P<0.01;

***, P<0.001 compared with wild-type mice;

#, P<0.05;

##, P<0.01;

###, P<0.001 compared with CaR^−/−^ mice.

To determine potential interactions between the effects of CaR and 1,25(OH)_2_D_3_ or PTH on parathyroid gland growth, the size of the parathyroid glands were examined by histology and image analysis. The parathyroid glands were mildly enlarged in CaR^−/−^ mice (Figure 1E–1H) and 1α(OH)ase^−/−^ mice (Figure 1E and 1G), and were significantly enlarged in PTH^−/−^ mice compared (Figure 1F and 1H) to the wild-type mice. Ablation of 1α(OH)ase in CaR^−/−^ mice resulted in further enlargement of the parathyroid glands (Figure 1E and 1G). Ablation of PTH in CaR^−/−^ mice also resulted in more marked enlargement of parathyroid glands, which, however, were only slightly more enlarged relative to PTH^−/−^ mice (Figure 1F and 1H).

### Effects of deletion of 1α(OH)ase or PTH on the skeletal development of CaR^−/−^ mice

**Figure 2:**
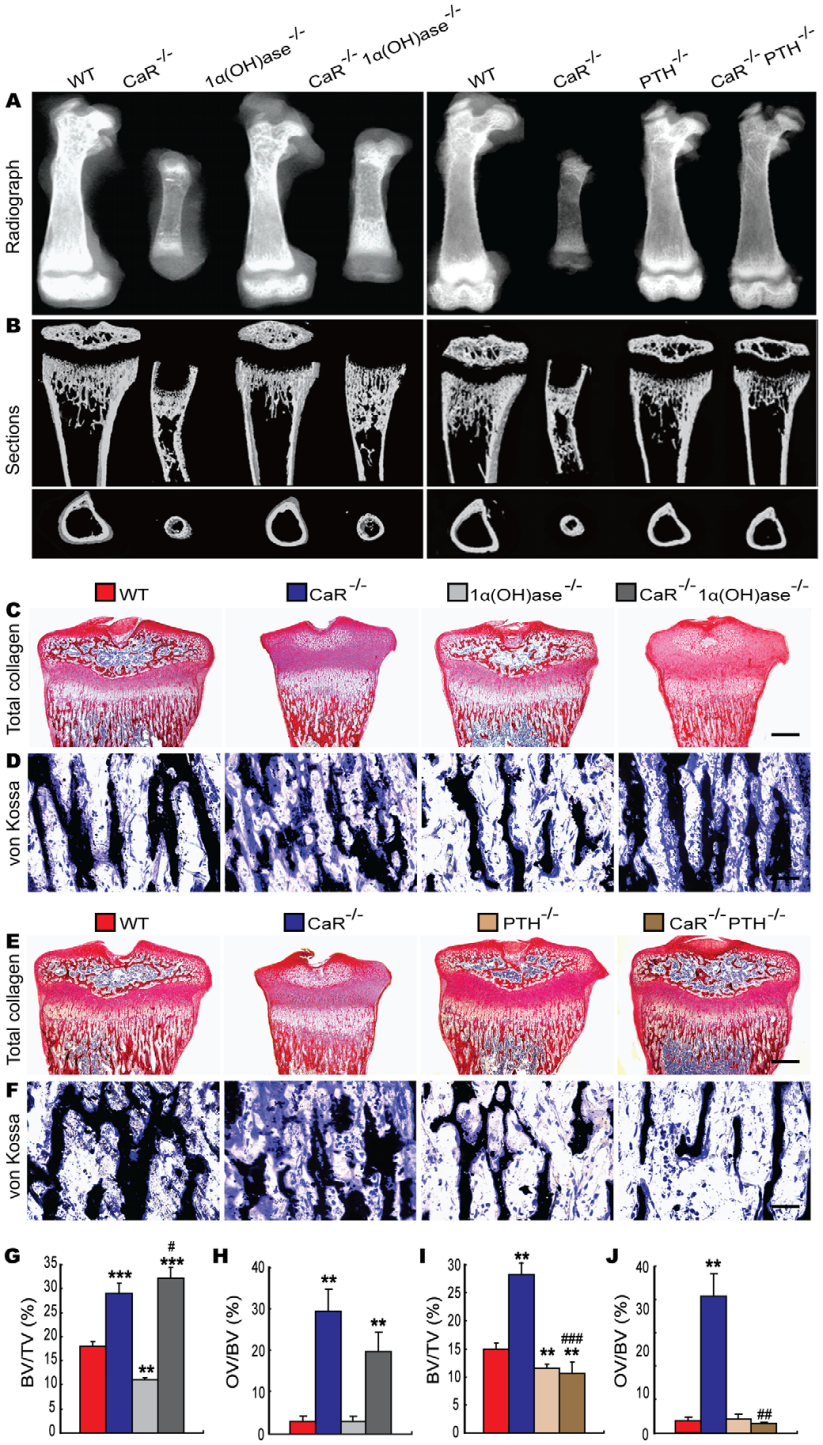
Effects of deletion of 1α(OH)ase or PTH on the skeletal development of CaR–deficient mice. (A) Representative contact radiographs of femurs from 2-week-old wild-type (WT), CaR^−/−^, 1α(OH)ase^−/−^, PTH^−/−^, CaR^−/−^1α(OH)ase^−/−^ and CaR^−/−^PTH^−/−^ mice. (B) Representative longitudinal and cross sections of three-dimensional reconstructed proximal ends of tibiae and mid shaft diaphyses utilizing micro-CT. (C, E) Representative micrographs of decalcified paraffin-embedded sections of the proximal ends of tibiae stained histochemically for total collagen. (D, F) Representative micrographs from undecalcified sections of the proximal ends of tibiae stained by the von Kossa procedure. Scale bars represent 200 µm in C and E and 25 µm in D and F. (G, I) Trabecular bone volume relative to tissue volume (BV/TV, %). (H, J) Osteoid volume relative to bone volume (OV/BV, %). Each value is the mean±SEM of determinations in 6 mice of the same genotype. **, P<0.01; ***, P<0.001 compared with wild-type mice; #, P<0.05; ##, P<0.01; ###, P<0.001 compared with CaR^−/−^ mice.

To assess the interaction between CaR and 1,25(OH)_2_D_3_ or PTH on skeletal development, skeletal phenotypes of gender-matched wild-type and the five mutant models were analyzed at 2 weeks of age by radiography, micro-CT and histology. Radiographs of femurs demonstrated that the lengths of femurs were dramatically reduced in CaR^−/−^ mice, while those of the 1α(OH)ase^−/−^ and PTH^−/−^ mice were only slightly shortened compared with their wild-type littermates (Figure 2A). Ablation of 1α(OH)ase in CaR^−/−^ mice resulted in an increase in the lengths of femurs, but they were still shorter than in wild-type mice, whereas ablation of PTH in CaR^−/−^ mice resulted in normalization of the lengths of femurs (Figure 2A). Radiolucency in metaphyses and diaphyses was increased markedly and no mineralized epiphyses were detected in CaR^−/−^ mice. Radiolucency in whole femurs was slightly increased in 1α(OH)ase^−/−^ and PTH^−/−^ mice. Ablation of 1α(OH)ase in CaR^−/−^ mice reduced the radiolucency, especially in the metaphyses, and ablation of PTH in CaR^−/−^ mice also reduced the radiolucency relative to CaR^−/−^ mice; however, the radiolucency was still greater than that in their wild-type littermates (Figure 2A).

From longitudinal and cross sections of three-dimensional reconstructed proximal ends of tibiae and middle diaphyses (Figure 2B), it can been seen that mineralized trabecular bone volume in metaphyses was increased in CaR^−/−^ mice, although no mineralized epiphyses were detected, and mineralized cortical bone volume was reduced in these mice. Mineralized epiphyseal volume, cortical and trabecular volume were reduced and the width of unmineralized growth plates was greater in 1α(OH)ase^−/−^ mice. Mineralized cortical and trabecular bone volumes were reduced in PTH^−/−^ mice. Ablation of 1α(OH)ase in CaR^−/−^ mice resulted in a marked increase in mineralized trabecular bone volume, whereas ablation of PTH in CaR^−/−^ mice nearly normalized mineralization in epiphyses, although mineralized cortical and trabecular bone volumes were still lower than in their wild-type littermates (Figure 2B). Longitudinal sections of proximal ends of tibiae stained histochemically for total collagen are shown in Figure 2C and 2E, and trabecular volume was measured (Figure 2G and 2I). Trabecular bone volume was increased significantly in CaR^−/−^ mice and reduced in 1α(OH)ase^−/−^ and PTH^−/−^ mice relative to their wild-type littermates. Ablation of 1α(OH)ase in CaR^−/−^ mice resulted in a more dramatic increase in the trabecular bone volume, whereas ablation of PTH in CaR^−/−^ mice resulted in a marked reduction in the trabecular bone volume, even compared to PTH^−/−^ mice (Figure 2C, 2E, 2G and 2I). Osteoid volume was increased significantly in CaR^−/−^ and CaR^−/−^1α(OH)ase^−/−^ mice, and was not altered significantly in 1α(OH)ase^−/−^, PTH^−/−^ and CaR^−/−^PTH^−/−^ mice relative to their wild-type littermates (Figure 2D, 2F, 2H and 2J).

### Effects of deletion of 1α(OH)ase or PTH on endochondral bone formation in CaR^−/−^ mice

**Figure 3:**
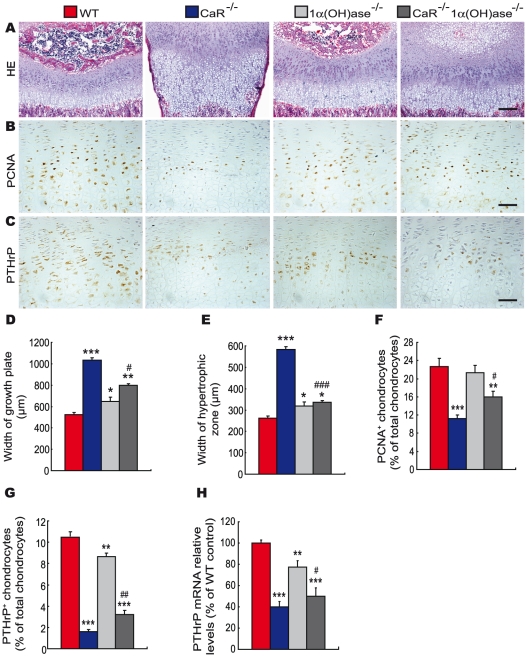
Effects of deletion of 1α(OH)ase on endochondral bone formation in CaR–deficient mice. Paraffin-embedded sections of tibiae from 2-week-old wild-type (WT), CaR^−/−^, 1α(OH)ase^−/−^ and CaR^−/−^1α(OH)ase^−/−^ mice stained (A) with HE, (B) immunohistochemically for PCNA, and (C) immunohistochemically for PTHrP. Scale bars in A, B and C represent 100, 25 and 25 µm. Width of (D) growth plates and (E) hypertrophic zones of growth plates. (F) The percentage of PCNA-positive chondrocytes relative to total chondrocytes. (G) The percentage of PTHrP-positive chondrocytes relative to total chondrocytes. (H) Comparison of PTHrP gene expression levels in growth plates of WT, CaR^−/−^, 1α(OH)ase^−/−^ and CaR^−/−^1α(OH)ase ^−/−^ mice. Specific PTHrP products were amplified from the tissue RNAs by real-time RT–PCR as described in Materials and Methods. Messenger RNA expression assessed by real-time RT–PCR analysis was calculated as a ratio to the GAPDH mRNA level and expressed relative to levels of WT mice. Each value is the mean±SEM of determinations in 6 mice of the same genotype. *, P<0.05; **, P<0.01; ***, P<0.001 compared with wild-type mice; #, P<0.05; ##, P<0.01; ###, P<0.001 compared with CaR^−/−^ mice.

**Figure 4:**
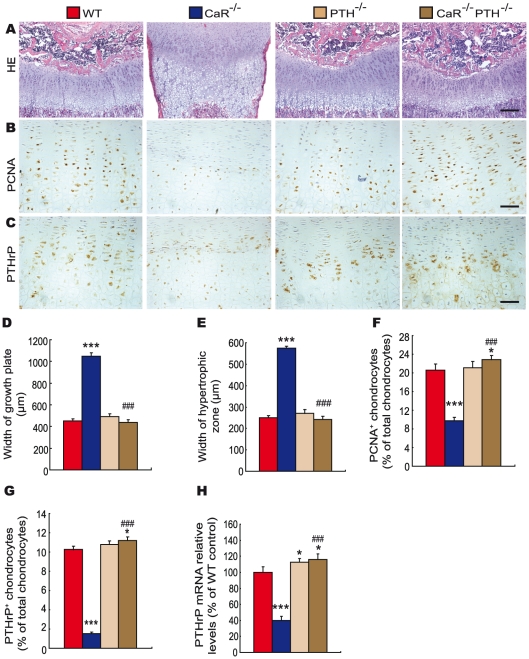
Effects of deletion of PTH on endochondral bone formation in CaR–deficient mice. Paraffin-embedded sections of tibiae from 2-week-old wild-type (WT), CaR^−/−^, PTH^−/−^ and CaR^−/−^PTH^−/−^ mice stained (A) with HE, (B) immunohistochemically for PCNA and (C) immunohistochemically for PTHrP. Scale bars in A, B and C represent 100, 25 and 25 µm. Width of (D) growth plates and (E) hypertrophic zones of growth plates. (F) The percentage of PCNA-positive chondrocytes relative to total chondrocytes. (G) The percentage of PTHrP-positive chondrocytes relative to total chondrocytes. (H) Comparison of PTHrP gene expression levels in growth plates of WT, CaR^−/−^, PTH^−/−^ and CaR^−/−^PTH^−/−^ mice. Specific PTHrP products were amplified from the tissue RNAs by real-time RT–PCR as described in Materials and Methods. Messenger RNA expression assessed by real-time RT–PCR analysis was calculated as a ratio to the GAPDH mRNA level and expressed relative to levels of WT mice. Each value is the mean±SEM of determinations in 6 mice of the same genotype. *, P<0.05; ***, P<0.001 compared with wild-type mice; ###, P<0.001 compared with CaR^−/−^ mice.

To assess the interaction between CaR and 1,25(OH)_2_D_3_ or PTH on endochondral bone formation, the phenotypes of growth plates were analyzed at 2-weeks of age by histology and immunohistochemistry. Only a few hypertrophic chondrocytes were observed in secondary ossification centers, and the width of growth plates and hypertrophic zones were increased dramatically in CaR^−/−^ mice, increased in 1α(OH)ase^−/−^ mice and not altered in PTH^−/−^ mice relative to their wild-type littermates. Ablation of 1α(OH)ase in CaR^−/−^ mice accelerated slightly the formation of secondary ossification centers, resulted in more hypertrophic chondrocytes and the appearance of some osteoblasts and the width of growth plates and hypertrophic zones were reduced significantly. Ablation of PTH in CaR^−/−^ mice normalized the secondary ossification centers and the growth plates (Figure 3A, 3D, 3E and Figure 4A, 4D, 4E).

The proliferation of chondrocytes was assessed by immunostaining for proliferating cell nuclear antigen (PCNA), and the percentage of PCNA-positive chondrocytes was quantitated. The percentage of PCNA-positive chondrocytes was reduced dramatically in CaR^−/−^ mice, and was not altered significantly in 1α(OH)ase^−/−^ and PTH^−/−^ mice. Ablation of 1α(OH)ase in CaR^−/−^ mice increased significantly the percentage of PCNA-positive chondrocytes; however, it was still lower than in their wild-type littermates. Ablation of PTH in CaR^−/−^ mice increased the percentage of PCNA-positive chondrocytes more dramatically, to a level even higher than that in their wild-type littermates (Figure 3B, 3F and Figure 4B, 4F).

In view of the fact that PTHrP plays an important role in the proliferation and apoptosis of chondrocytes, we examined the localization of PTHrP in chondrocytes by immunostaining and the expression of the PTHrP gene in cartilaginous growth plates. The percentage of PTHrP-positive chondrocytes was reduced markedly in CaR^−/−^ mice, reduced significantly in 1α(OH)ase^−/−^ mice and increased in PTH^−/−^ mice. Ablation of 1α(OH)ase in CaR^−/−^ mice increased significantly the percentage of PTHrP-positive chondrocytes; however, it was still lower than in their wild-type littermates. Ablation of PTH in CaR^−/−^ mice increased markedly the percentage of PTHrP-positive chondrocytes (Figure 3C, 3G and Figure 4C, 4G). Alteration in the pattern of expression of the PTHrP gene in cartilaginous growth plates were similar to those observed for the percentage of PTHrP positive chondrocytes (Figure 3H and Figure 4H).

### Effects of deletion of 1α(OH)ase or PTH on osteoblastic bone formation in CaR^−/−^ mice

**Figure 5:**
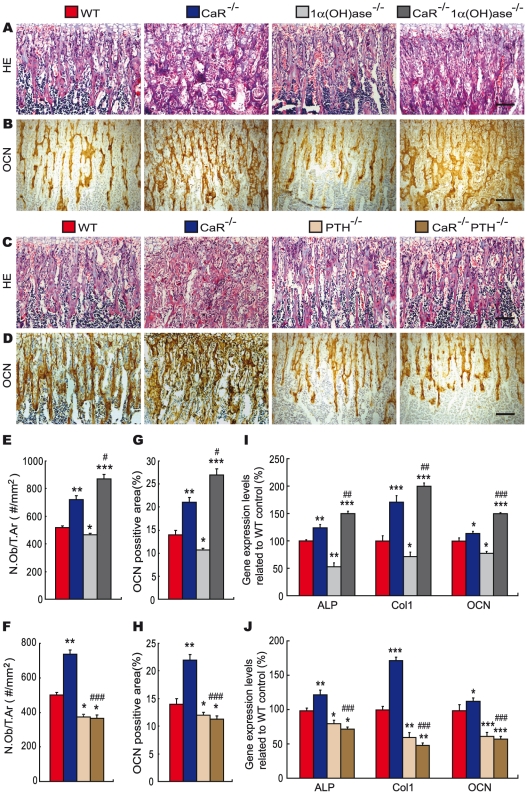
Effects of deletion of 1α(OH)ase or PTH on osteoblastic bone formation in CaR–deficient mice. Representative micrographs of paraffin-embedded sections of tibiae from 2-week-old wild-type (WT), CaR^−/−^, 1α(OH)ase^−/−^, PTH^−/−^, CaR^−/−^1α(OH)ase^−/−^ and CaR^−/−^PTH^−/−^ mice stained (A, C) with HE, (B, D) or immunohistochemically for osteocalcin (OCN). Scale bars represent 50 µm in A–D. (E, F) Number of osteoblasts per tissue area (N.Ob/T.Ar, #/mm^2^). (G, H) Osteocalcin-positive area as a percent of the tissue area. (I, J) Real-time RT–PCR of long bone tissue extracts for the expression of ALP, type I collagen (Col I) and OCN. Messenger RNA expression assessed by real-time RT–PCR is calculated as a ratio to the GAPDH mRNA level and expressed relative to levels of WT mice. Each value is the mean ± SEM of determinations in 6 animals of the same genotype. *, P<0.05; **, P<0.01; ***, P<0.001 compared with wild-type mice; #, P<0.05; ##, P<0.01; ###, P<0.001 compared with CaR^−/−^ mice.

To determine whether alterations in trabecular bone volume were associated with those in osteoblastic bone formation, paraffin sections from the various genotypes of mice at 2 weeks of age were stained with HE (Figure 5A and 5C) as well as immunohistochemically for osteocalcin (Figure 5B and 5D). The number of osteoblasts (Figure 5E and 5F) and osteocalcin-positive areas (Figure 5G and 5H) in metaphyseal regions were quantitated by image analysis. The number of osteoblasts and osteocalcin-positive areas were increased significantly in CaR^−/−^ mice, while they were reduced in 1α(OH)ase^−/−^ and PTH^−/−^ mice relative to their wild-type littermates. Ablation of 1α(OH)ase in CaR^−/−^ mice resulted in a more dramatic increase in the number of osteoblasts and osteocalcin-positive areas, whereas ablation of PTH in CaR^−/−^ mice resulted in a marked reduction in the number of osteoblasts and osteocalcin-positive areas, even compared to PTH^−/−^ mice (Figure 5A–5H). We also examined alterations in the expression of genes related to bone formation. RNA was isolated from long bones and real-time RT–PCR was performed. Results showed that the expression of the ALP, type I collagen and osteocalcin genes were increased significantly in CaR^−/−^ mice, while they were reduced in 1α(OH)ase^−/−^ and PTH^−/−^ mice relative to their wild-type littermates. Ablation of 1α(OH)ase in CaR^−/−^ mice resulted in a more dramatic increase in the expression of these same genes, whereas ablation of PTH in CaR^−/−^ mice resulted in a marked reduction in their expression, even compared to PTH^−/−^ mice (Figure 5I and 5J). These alterations were consistent with those of osteoblastic bone formation parameters observed by histomorphometric analysis.

### Effects of deletion of 1α(OH)ase or PTH on osteoclastic bone resorption in CaR^−/−^ mice

**Figure 6:**
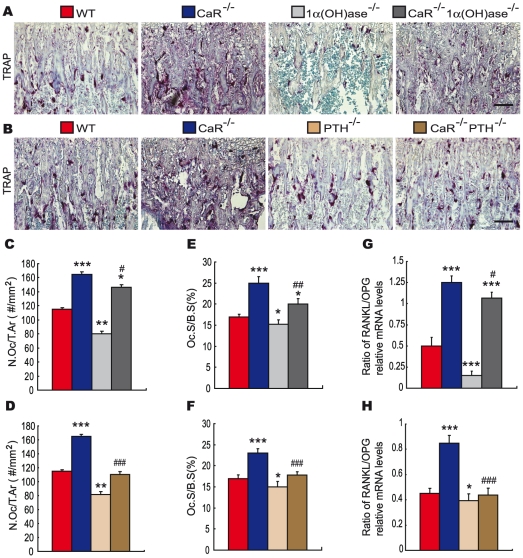
Effects of deletion of 1α(OH)ase or PTH on osteoclastic bone resorption in CaR–deficient mice. (A, B) Representative micrographs of paraffin-embedded sections of tibiae from 2-week-old wild-type (WT), CaR^−/−^, 1α(OH)ase^−/−^, PTH^−/−^, CaR^−/−^1α(OH)ase^−/−^ and CaR^−/−^PTH^−/−^ mice stained histochemically for tartrate-resistant acid phosphatase activity (TRAP). Scale bars in A and B represent 50 µm. (C, D) Number of TRAP-positive osteoclasts per tissue area (N.Oc/T.Ar, #/mm^2^) and (E, F) the surface of osteoclasts relative to the bone surface (Oc.S/B.S, %). Each value is the mean ± SEM of determinations in 6 animals in each group. (G, H) Real-time RT–PCR was performed on long bone tissue extracts for RANKL and OPG mRNA as described in Materials and Methods. Messenger RNA expression assessed by real-time RT–PCR analysis is calculated as a ratio to the GAPDH mRNA level and expressed relative to levels of wild-type mice. Ratio of RANKL/OPG relative mRNA levels was calculated. Each value is the mean ± SEM of determinations in 6 animals of the same genotype. *, P<0.05; **, P<0.01; ***, P<0.001 compared with wild-type mice; #, P<0.05; ##, P<0.01; ###, P<0.001 compared with CaR^-/-^ mice.

To assess the interaction between CaR and 1,25(OH)_2_D_3_ or PTH on osteoclastic bone resorption, paraffin sections were stained histochemically for TRAP (Figure 6A and 6B). Osteoclast number (Figure 6C and 6D) and surface (Figure 6E and 6F) were determined by image analysis. The results revealed that the TRAP-positive osteoclast number and surface were increased markedly in the metaphyseal regions in CaR^−/−^ mice and were decreased significantly in 1α(OH)ase^−/−^ and PTH^−/−^ mice relative to their wild-type littermates. Ablation of 1α(OH)ase in CaR^−/−^ mice decreased significantly osteoclast number and surface; however, these parameters were still greater than in their wild-type littermates. Ablation of PTH in CaR^−/−^ mice markedly decreased osteoclast number and surface (Figure 6A–6F). We also examined alterations in the expression of genes related to bone resorption. RNA was isolated from long bones. The gene expression of the RANKL and OPG genes were examined by real-time RT–PCR, and the ratio of RANKL/OPG mRNA levels was calculated. Results revealed that alterations in the ratio of RANKL/OPG mRNA levels were consistent with those of TRAP-positive osteoclast number and surface (Figure 6G and 6H).

## Discussion

In the present study, we examined the effect on mineral homeostasis of deleting CaR (CaR^−/−^) alone, or both 1,25(OH)_2_D_3_ and CaR (1α(OH)ase^−/−^CaR^−/−^), or both PTH and CaR (PTH^−/−^CaR^−/−^). Our results confirmed previous findings in CaR^−/−^ mice: namely the presence of hypercalcemia, hypophosphatemia, hyperparathyroidism and enlarged parathyroid glands [5], [6]. Deletion of 1α(OH)ase in the CaR null background corrects the hypercalcemia but leads to more severe hypophosphatemia, hyperparathyroidism and much larger parathyroid glands. These alterations were associated with diminished capacity of double knockout mice to stimulate absorption of calcium and phosphate from the gastrointestinal tract and to repress PTH biosynthesis, and resulted in a further stimulus for parathyroid gland enlargement, overproduction of parathyroid hormone and consequent effects on impeding renal phosphate reabsorption. Interestingly, deletion of PTH in CaR^−/−^ mice also corrects the hypercalcemia, and the mice develop hyperphosphatemia and undetectable serum PTH with enlarged parathyroid glands. These alterations were associated with diminished capacity of double mutant animals to stimulate calcium reabsorption and to inhibit phosphate reabsorption from the renal tubules [23].

Reduction of hypercalcemia in the CaR^−/−^ mice was the common biochemical alteration induced by deleting the 1α(OH)ase in the CaR^−/−^1α(OH)ase^−/−^ mice and PTH in the CaR^−/−^PTH^−/−^ mice. Improvement in hypercalcemia is therefore likely to have been the major contributor to the improved longevity of the CaR^−/−^ mice.

Previous studies have demonstrated that 1,25(OH)_2_D_3_ acts via the nuclear vitamin D receptor (VDR) to bind to a negative response element tentatively identified in the PTH promoter, which can repress PTH gene expression [17]. We previously showed that 1,25(OH)_2_D_3_ can inhibit the growth of primary cultures of bovine parathyroid cells in vitro [24]. We also showed that 1,25(OH)_2_D_3_ inhibits parathyroid hyperplasia in vivo in a mouse model with targeted ablation of the 1α(OH)ase gene [25]. Our current study found more severe hypophosphatemia and hyperparathyroidism with enlarged parathyroid glands in 2-week-old double homozygous CaR- and 1α(OH)ase-deficient neonates than in CaR^−/−^ mice, indicating that 1,25(OH)_2_D_3_ inhibits parathyroid growth and PTH biosynthesis in a CaR-independent manner.

We next examined the effect of CaR deficiency and the interaction between CaR and 1,25(OH)_2_D_3_ or PTH on endochondral bone formation. Our results show that CaR deficiency resulted in a severe delay of secondary ossification center formation, an enlarged growth plate and a significant reduction of chondrocyte proliferation and apoptosis. Genetic deficiencies in 1,25(OH)_2_D_3_ caused by loss of a functional 1α(OH)ase enzyme or vitamin D receptor (VDR) result in hypocalcemia, hyperparathyroidism, hypophosphatemia, rickets, and osteomalacia. These effects, however, are very mild or absent in suckling mice but become markedly accelerated in mice after weaning, when dietary calcium and lactose content decreases. The phenotype can largely be corrected by administration of a high calcium, high lactose-containing diet that bypasses active, 1,25(OH)_2_D_3_-mediated, Ca^2+^ absorption [25]. It is noteworthy that analysis of the chondrocyte-specific Cyp27b1-knockout and Cyp27b1-overexpressing mice revealed a fetal bone phenotype which did not persist however beyond the immediate neonatal period [26]. Consequently although 1,25(OH)_2_D_3_ may play a direct role in normal development of the cartilaginous growth plate, other factors, such as endocrine maintenance of calcium and phosphate balance may be more important in defining postnatal bone development. The deletion of 1α(OH)ase in CaR^−/−^ mice slightly accelerated secondary ossification center formation, and improved chondrocyte proliferation, while deletion of PTH in CaR^−/−^ mice rescued these abnormalities of cartilaginous development in CaR^−/−^ mice. This result is consistent with a previous report in which correction of severe hyperparathyroidism in CaR^−/−^ mice resulted in healing of the rickets and osteomalacia [11]. In our studies, the normalization of serum calcium in both double mutants may have accelerated the improvements in the secondary ossification centre and in the cartilaginous growth plate.

Previous studies have reported that the CaR modulates growth plate chondrocyte differentiation in vitro [27]–[29], and chondrocyte-specific CaR deletion leads to early embryonic death (before E13). However, if the deletion occurs at the later time points, such as between E16 and E18, viable pups can be obtained but with delayed growth plate development [7]. Currently, there is no fully accepted explanation for these discrepancies in phenotype between chondrocyte-specific CaR knockout mice obtained using mice with a floxed exon 7 of the CaR gene [7] and the CaR conventional knockout mice in which exon 5 has been disrupted [6], [10]. Because calcium regulation is so fundamental for the maintenance of normal cell function, it is plausible that cells have some other pathways that can compensate for neonatal defects resulting from deficiency of CaR. One such compensatory mechanism may be the expression of a CaR splice variant lacking exon 5 in chondrocytes from the CaR–deficient mice [30].

In the PTH^−/−^CaR^−/−^ mice complete rescue of the alterations in the secondary ossification centre and in the cartilaginous growth plate were observed. In view of the fact that PTH is not synthesized in cartilage, it cannot access this avascular tissue. It seems likely that, since phosphate per se plays a critical role in growth plate development (30), and mineralization [31] and phosphate levels are also markedly decreased in CaR^−/−^ mice and increased by deletion of PTH, hyperphosphatemia in the CaR^−/−^PTH^−/−^ mice likely contributed to the improvement in the growth plate.

PTHrP-deficient mice display reduced chondrocyte proliferation, accelerated differentiation, and increased apoptosis [31], [32]. Our previous study demonstrated that 4-month old PTH^−/−^ mice have significantly increased serum PTHrP concentrations and PTHrP mRNA and protein levels in bone tissues, suggesting that PTHrP is required for the increased trabecular bone volume observed in adult PTH^−/−^ mice [33]. Based on these findings, we hypothesized that altered PTHrP levels might account for the skeletal defects of CaR^−/−^ mice and play a compensatory role in PTH/CaR double knockout mice. To test this hypothesis, we examined the levels of expression of PTHrP mRNA and protein in cartilaginous growth plates by real-time RT-PCR and immunohistochemistry, respectively. PTHrP-immunopositive chondrocytes and PTHrP mRNA levels were markedly decreased in CaR^−/−^ mice, slightly increased in CaR^−/−^1α(OH)ase^−/−^ mice, and increased significantly in CaR^−/−^PTH^−/−^ mice compared to their wild-type littermates. These results demonstrate that down-regulation of PTHrP expression in chondrocytes is associated with defects in the growth plate caused by CaR deficiency and that up-regulation of PTHrP expression is associated with either improvement or rescue of chondrocyte abnormalities resulting from the deletion of either 1α(OH)ase or PTH, in the CaR–deficient mice. 1,25(OH)_2_D_3_ has been reported to dampen PTHrP upregulation at both the mRNA and protein levels in prostate cancer cells [34], [35]. 1,25(OH)_2_D_3_ deficiency may therefore have contributed to the increases of PTHrP observed in the double mutants. Taken together, these data suggest that the down-regulation of PTHrP expression in chondrocytes may contribute to defects of cartilage in the CaR–deficient neonates and that up-regulation of PTHrP expression in chondrocytes may exert a contributory role in rescuing these abnormalities.

We also examined the effect of 1,25(OH)_2_D_3_ or PTH deficiency in CaR^−/−^ mice on osteoblastic bone formation. CaR–deficient mice showed increased trabecular volume, osteoblast number, and osteocalcin-positive areas, as well as increased expression of the ALP, type I collagen and osteocalcin genes and higher serum ALP levels. These osteoblastic bone formation parameters increased dramatically in 1α(OH)ase/CaR double knockout mice, despite the fact that these parameters were slightly decreased in 1α(OH)ase-deficient neonates. These results are consistent with our previous findings from 2-week-old [22] and adult 1α(OH)ase^−/−^ mice [19], [25]. When the phenotype of 1α(OH)ase^−/−^ mice was analyzed, we found that the skeletal phenotype was different before and after weaning. In 2-week-old 1α(OH)ase^−/−^ mice, the trabecular volume and osteoblast numbers were decreased, and the osteoid volume was not increased significantly [22]. In contrast, in 4-month-old 1α(OH)ase^−/−^ mice, the trabecular volume, osteoblast number and osteoid volume were all increased significantly even on a high calcium diet containing 1.5% calcium in the drinking water [25]. These differences were thought to result from the elevations in circulating PTH. Breast-feeding of neonatal 1α(OH)ase^−/−^ pups with milk containing a higher calcium and low level of 1,25(OH)_2_D_3_ had less severe hypocalcemia and subsequent less elevation of serum PTH compared to adult mutant mice. Our previous studies had shown that serum PTH was increased 1.5-fold at 2 weeks of age [22], but 30-fold at 4 months of age [25], in the 1α(OH)ase^−/−^ mice compared to their wild-type counterparts. If the stimulatory effects of elevated PTH on osteoblasts could not overcome the reduction in osteoblasts due to 1,25(OH)_2_D_3_ deficiency, osteoblastic bone formation parameters would be reduced. If, on the other hand, the osteoblast-stimulating effects of elevated PTH overcame the decreased osteoblasts resulting from 1,25(OH)_2_D_3_ deficiency, then osteoblastic bone formation parameters would be increased. The presence of increased numbers of osteoblasts and increased bone matrix volume in CaR^−/−^ and CaR^−/−^1α(OH)ase^−/−^ neonates suggests that any requirement of either CaR or/and 1,25(OH)_2_D_3_ for osteoblast activation is readily overcome by the osteoblast stimulating effects of elevated PTH. This observation was further supported by deletion of PTH in the CaR^−/−^ mice, which resulted in a significant decrease in osteoblastic bone formation compared to PTH^−/−^ mice. The difference in osteoblastic bone formation between the PTH^−/−^ and CaR^−/−^PTH^−/−^ mice and which can be accentuated in older animals [36] is consistent with a stimulatory effect of CaR on osteoblast function. Indeed, exposure of primary osteoblasts or a variety of osteoblast-like cells to high calcium or polycationic CaR agonists, such as neomycin and gadolinium, stimulate their proliferation, differentiation and mineralization [37], [38].

Previous studies have revealed that calcium is also an important regulator of osteoclast function. Exposing osteoclasts to high extracellular calcium concentrations results in dramatic cell retraction followed by profound inhibition of bone resorption [39]–[41]. Subsequent studies found that very high calcium inhibits the bone-resorbing activity of osteoclasts by directly acting on the CaR that is present in osteoclast precursor cells [42] and mature osteoclasts [43], [44]. Our results found that TRAP-positive osteoclast number and surface and the ratio of RANKL/OPG were increased in CaR^−/−^ mice. Deletion of 1α(OH)ase in CaR^−/−^neonates resulted in a decrease in osteoclastic bone resorption parameters, however, these parameters were higher than in 1α(OH)ase neonates. Deletion of PTH in CaR^−/−^ neonates also resulted in a decrease in these parameters. As in our previous report [22], the current study confirms that osteoclastic bone resorption parameters were reduced in either 1α(OH)ase^−/−^ or PTH^−/−^ neonates. Taken together, these studies suggest that the CaR, 1,25(OH)_2_D_3_ and PTH are all required for osteoclastic bone resorption.

Results from this study indicate that normocalcemia in patients with NSHPT may lengthen their lifespan, and deletion of PTH in patients with NSHPT may normalize skeletal growth and development. A previous study has reported that the use of intravenous pamidronate controlled severe hypercalcaemia in NSHPT patients prior to parathyroidectomy [45], Consequently, they recommended the short-term use of pamidronate in neonatal severe hyperparathyroidism to treat extreme hypercalcaemia and halt hyperparathyroid-driven skeletal demineralization in preparation for parathyroidectomy. Recently, a retrospective review has been conducted for patients managed for NSHPT over the last 10 years [46]. Five patients with NSHPT, 3 females and 2 males, presented at a mean age of 18 days. All patients had a total parathyroidectomy and autotransplantation at a mean age of 65 days, with a mean follow-up of 5.5 years. One patient had normal parathyroid hormone and normal calcium levels 9.5 years after surgery without medication. One patient had normal levels without medication for 2 years then needed calcium and vitamin D supplements thereafter (8.5 years postoperatively). Three patients are still on calcium and vitamin D supplementation at 5.5 years, 3.5 years, and 8 months, respectively, after surgery. Consequently, they conclude that NSHPT is managed effectively with total parathyroidectomy [46].

In summary (Table 2), CaR^−/−^ mice had a very short lifespan, decreased body weight and displayed hypercalcemia, hypophosphatemia, elevated serum ALP, PTH and 1,25(OH)_2_D_3_; CaR^−/−^1α(OH)ase^−/−^ mice had slightly increased lifespan and body weight and displayed normocalcemia, hypophosphatemia, greater elevations in PTH and ALP, and undetectable serum 1,25(OH)_2_D_3_; CaR^−/−^PTH^−/−^ mice displayed normocalcemia, hyperphosphatemia, normal ALP levels, undetectable serum PTH and lower serum 1,25(OH)_2_D_3_. CaR deficiency resulted in a severe delay of secondary ossification center formation, an enlarged growth plate and a significant reduction of chondrocyte proliferation. These alterations were associated with hypophosphatemia and decreased PTHrP expression in chondrocytes. Deletion of 1α(OH)ase in CaR^−/−^ mice partially rescued the cartilage phenotype associated with an increase in PTHrP expression in chondrocytes but persistent hypophosphatemia. Deletion of PTH in CaR^−/−^ mice rescued the phenotype of cartilage associated with the up-regulation of PTHrP expression in chondrocytes and correction of hypophosphatemia. The alterations of bone formation parameters were consistent with elevated serum PTH and with the reduction of PTHrP gene expression in bony tissue in CaR^−/−^ and CaR^−/−^ 1α(OH)ase^−/−^ mice. Deletion of PTH in CaR^−/−^ mice resulted in a significant decrease in osteoblastic bone formation, which was not rescued completely by increased PTHrP gene expression in bony tissue. Osteoclastic bone resorption parameters were increased in CaR^−/−^mice, however, they were decreased in CaR^−/−^1α(OH)ase^−/−^ mice and more dramatically in CaR^−/−^PTH^−/−^ mice compared to CaR^−/−^ littermates. The current study demonstrates that (1) hypercalcemia, contributes to the early lethality of CaR–deficient mice, (2) defects in endochondral bone formation in CaR–deficient mice result from effects of the excess elevations in calcium and the decrease in phosphorus and PTHrP levels, while (3) the increased osteoblastic bone formation results from direct effects of PTH. Our results therefore provide mechanistic insight into the improvement in longevity and normalization in skeletal growth and development observed in patients with NSHPT after total parathyroidectomy.

**Table 2:** Summary of interactions among CaR, 1,25(OH)_2_D_3_, and PTH on mineral and skeletal homeostasis.

	CaR^−/−^	CaR^−/−^1α(OH)ase^−/−^	CaR^−/−^PTH^−/−^
**Changes of lifespan and bone weight**			
Lifespan	↓↓↓	↓↓	→
Body weight	↓↓↓	↓↓	→
**Mineral ion homeostasis**			
Serum calcium	↑↑↑	→	→
Serum phosphorus	↓↓	↓↓↓	↑
Serum ALP	↑↑	↑↑↑	→
Serum PTH	↑↑	↑↑↑	**—**
Serum 1,25(OH)_2_D_3_	↑↑↑	**—**	↓
**Cartilage changes**			
Secondary ossification center	↓↓↓	↓↓	→
Width of growth plate	↑↑↑	↑	→
Chondrocyte proliferation	↓↓↓	↓↓	↑
PTHrP^+^ chondrocytes	↓↓↓	↓↓	↑
**Bone changes**			
Trabecular bone volume	↑↑	↑↑↑	↓
Osteoblast production	↑↑	↑↑↑	↓
Bone mineralization	↓↓↓	↓↓	→
Osteoclast production	↑↑	↑	→

↓, a reduction; ↑, an increase; →, no change. and **—**, undetectable. Repetitive arrows indicate the magnitude of the change.

## Materials and Methods

### Derivation of CaR/1α(OH)ase double null mice and CaR/PTH double null mice

The derivation of the three parental strains of CaR^+/−^, PTH^+/−^mice and 1α(OH)ase^+/−^ mice by homologous recombination in embryonic stem cells was previously described by Ho et al. [5], Miao et al. [8], [9] and Panda et al.[19], respectively. Briefly, a neomycin resistance gene was inserted into exon III of the mouse CaR gene or into exon III of the mouse *Pth* gene, replacing a portion of the extracellular domain of the CaR, which senses extracellular calcium, and the entire coding sequence of the PTH gene. Lack of CaR and PTH expression were confirmed by Western blot of kidney protein membrane extracts from homozygous CaR^−/−^ mice [5] and by immunostaining of parathyroid glands sections [8], respectively. A neomycin resistance gene replaced exons VI, VII, and VIII of the mouse 1α(OH)ase gene, removing both the ligand-binding and the heme-binding domains. RT–PCR of renal RNA from homozygous 1α(OH)ase^−/−^ mice confirmed the lack of 1α(OH)ase expression [19]. CaR^+/−^ and 1α(OH)ase^+/−^ mice were fertile and were mated to produce offspring heterozygous at both loci, which were then mated to generate CaR^−/−^1α(OH)ase^−/−^ pups. CaR^−/−^PTH^−/−^ pups were generated in the same way as above. Lines were maintained by mating CaR^+/−^1α(OH)ase^+/−^ males and females. These mice were of mixed genetic background, with contributions from C57BL/6J/129/SvJ and 129/SvEv/BALB/c strains. Mutant mice and control littermates were maintained in a virus- and parasite-free barrier facility and exposed to a 12-h/12-h light/dark cycle, and were fed a regular rodent diet. The use of animals in this study was approved by the Institutional Animal Care and Use Committee of Nanjing Medical University (Approval ID 2008-00518).

### Genotyping of mice

Tail fragment genomic DNA was isolated by standard phenol–chloroform extraction and isopropanol precipitation. To determine the genotype at the CaR, PTH and 1α(OH)ase loci, six PCR amplification reactions were conducted. To assay the presence of the wild-type *CaR* allele, samples were amplified with CaR forward primer (5′-TCT GTT CTC TTT AGG TCC TGA AAC A-3′) and CaR reverse primer (5′-TCA TTG ATG AAC AGT CTT TCT CCC T-3′). To detect the presence of the null *CaR* allele, Neo forward primer r-Neo-2 (5′-TCT TGA TTC CCA CTT TGT GGT TCT A-3′) was used with the CaR reverse primer. The presence of the wild-type *Pth* allele was detected using PTH forward primer (5′-GAT GTC TGC AAA CAC CGT GGC TAA-3′) and PTH reverse primer (5′-TCC AAA GTT TCA TTA CAG TAG AAG-3′). The null *Pth* allele was detected using Neo forward primer (5′-TCTTGATTCCCACTTTGTGGTTCTA-3′) and PTH reverse primer [10]. For the wild-type *Cyp27b1* [1α(OH)ase] allele, forward primer (5′-AGACTGCACTCCACTCTGAG-3′) and reverse primer (5′-GTT TCC TAC ACG GAT GTC TC-3′) were employed. The neomycin gene was detected with primers neo-F (5′-ACA ACA GAC AAT CGG CTG CTC-3′), and neo-R (5′-CCA TGG GTC ACG ACG AGA TC-3′) [25]. All PCR reactions were performed with 1 cycle of 95°C for 4 minutes, and 35 cycles of 94°C for 30 seconds, 55°C for 30 seconds, 72°C for 30 seconds.

### Biochemical and hormone analyses

Serum calcium and phosphorus were determined by autoanalyzer (Beckman Synchron 67; Beckman Instruments). Serum 1,25(OH)_2_D_3_ was measured by radioimmunoassay (ImmunoDiagnostic Systems, Bolden, UK) and intact PTH was measured by a two-site immunoradiometric assay (Immutopics, San Clemente, CA, USA).

### Radiography

Femurs were removed and dissected free of soft tissue. Contact radiographs were taken using a Faxitron model 805 radiographic inspection system (Faxitron Contact, Faxitron, Germany) (22 kV voltage and 4 min exposure time). X-Omat TL film (Eastman Kodak Co., Rochester, NY, USA) was used and processed routinely.

### Micro-computed tomography

Tibias obtained from 2-week-old mice were dissected free of soft tissue, fixed overnight in 70% ethanol and analyzed by micro-CT with a SkyScan 1072 scanner and associated analysis software (SkyScan, Antwerp, Belgium) as described [22]. Briefly, image acquisition was performed at 100 kV and 98 mA with a 0.98 rotation between frames. During scanning, the samples were enclosed in tightly fitting plastic wrap to prevent movement and dehydration. Thresholding was applied to the images to segment the bone from the background. Two-dimensional images were used to generate three-dimensional renderings using the 3D Creator software supplied with the instrument. The resolution of the micro-CT images is 11.26 microns.

### Histology

Thyroparathyroid tissue and tibias were removed and fixed in PLP fixative (2% paraformaldehyde containing 0.075 M lysine and 0.01 M sodium periodate) overnight at 4°C and processed histologically as described [47]. Tibias were decalcified in ethylene- diamine tetraacetic acid (EDTA)-glycerol solution for 5–7 days at 4°C. Decalcified bones and other tissues were dehydrated and embedded in paraffin after which 5 µm sections were cut on a rotary microtome. The sections were stained with hematoxylin and eosin (H&E) or histochemically for tartrate-resistant acid phosphatase (TRAP) activity and for total collagen or immunohistochemical staining for osteocalcin (OCN), parathyroid hormone-related protein (PTHrP) and proliferating cell nuclear antigen (PCNA) as described subsequently. Alternatively, undecalcified tibiae were embedded in LR White acrylic resin (London Resin Company Ltd., London, UK) and 1-µm sections were cut on an ultramicrotome. These sections were stained for mineral with the von Kossa staining procedure. Histomorphometric indices were determined as suggested by the ASBMR Histomorphometry Nomenclature Committee [48].

### Histochemical staining for total collagen and TRAP

Total collagen was detected in paraffin sections using a modified method of Lopez-De Leon and Rojkind [25]. Dewaxed sections were exposed to 1% sirius red in saturated picric acid for 1 h. After washing with distilled water, the sections were counterstained with hematoxylin and mounted with Biomount medium. Enzyme histochemistry for TRAP staining was performed as previously described [8]. Dewaxed sections were preincubated for 20 min in buffer containing 50 mM sodium acetate and 40 mM sodium tartrate at pH 5.0. Sections were then incubated for 15 min at room temperature in the same buffer containing 2.5 mg/ml naphthol AS-MX phosphate (Sigma-Aldrich, St. Louis, MO) in dimethylformamide as substrate and 0.5 mg/ml fast garnet GBC (Sigma-Aldrich) as a color indicator for the reaction product. After washing with distilled water, the sections were counterstained with methyl green and mounted in Kaiser's glycerol.

### Immunohistochemical staining

Osteocalcin, parathyroid hormone-related protein (PTHrP) and proliferating cell nuclear antigen (PCNA) were determined by immunohistochemistry using the avidin-biotin-peroxidase complex technique with an affinity-purified, goat anti-mouse osteocalcin antibody (Biomedical Technologies, Inc., Stoughton MA, USA), a mouse anti-PCNA monoclonal antibody (Medicorp Inc., Montreal, Canada) and a rabbit anti-serum against PTHrP[1]–[34], as described previously [8], [33], [47]. Briefly, the primary antibodies were applied to dewaxed paraffin sections overnight at room temperature. As a negative control, pre-immune serum was substituted for the primary antibody. After washing with high salt buffer (50 mM Tris–HCl, 2.5% NaCl, 0.05% Tween 20, pH 7.6) for 10 min at room temperature followed by two 10 min washes with TBS, the sections were incubated with secondary antibody (biotinylated goat anti-mouse IgG, Sigma), washed as before and incubated with the Vectastain Elite ABC kit (Vector Laboratories, Inc. Ontario, Canada) for 45 min. After washing as before, brown pigmentation to demarcate regions of immunostaining was produced by a 10–15 min treatment with the DAB kit (Vector Laboratories, Inc.). After washing with distilled water, the sections were counterstained with hematoxylin.

### Computer-assisted image analysis

Sections stained histochemically or immunohistochemically were photographed with a digital camera. Images of micrographs from single sections were digitally recorded using a rectangular template, and recordings were processed and analyzed using Northern Eclipse image analysis software as described [22], [47], [49]. Parathyroid gland size was quantitated in H & E stained sections using Northern Eclipse image analysis software and were presented as parathyroid areas (µm^2^).

### Quantitative real-time PCR

RNA was isolated from mouse bone tissues, using Trizol reagent (Invitrogen) according to the manufacturer's protocol. Reverse transcription reactions were performed using the SuperScript First-Strand Synthesis System (Invitrogen) as previously described [22], [33]. To determine the number of cDNA molecules in the reverse-transcribed samples, real-time PCR was performed using a LightCycler system (Roche Molecular Biochemicals, Indianapolis, IN, USA). PCR was performed using 2 µl LightCycler DNA Master SYBR Green I (Roche), 12.5 µl of reaction mixture, 2 µl of each 5′ and 3′ primer, 2 µl samples and then H_2_O was added to a final volume of 25 µl. Samples were denatured at 95°C for 10 sec, with a temperature transition rate of 20°C per sec. Amplification and fluorescence determination were carried out in four steps: denaturation at 95°C for 1 sec, with a temperature transition rate of 20°C /sec; annealing for 5 sec, with a temperature transition rate of 8°C /sec; extension at 72°C for 20 sec, with a temperature transition rate of 4°C /sec; and detection of SYBR Green fluorescence, which reflects the amount of double-stranded DNA, at 86°C for 3 sec. The amplification cycle number was 35. To discriminate specific from nonspecific cDNA products, a melting curve was obtained at the end of each run. Products were denatured at 95°C for 3 sec, and the temperature was then decreased to 58°C for 15 sec and raised slowly from 58 to 95°C using a temperature transition rate of 0.1°C /sec. To determine the number of copies of the targeted DNA in the samples, purified PCR fragments of known concentrations were serially diluted and served as external standards that were measured in each experiment. Data were normalized with GAPDH levels in the samples. The primer sequences used for the real-time PCR were the same as those used for routine PCR.

### Statistical analysis

Data from image analysis are presented as mean ± SEM. Statistical comparisons were made using a two-way ANOVA, with P<0.05 being considered significant.
